# Evaluation of aesthetic flat closure: A scoping review

**DOI:** 10.1016/j.jpra.2025.08.024

**Published:** 2025-08-24

**Authors:** Meghana Bhaskara, Luci Hulsman, Shahnur Ahmed, Angad S. Sidhu, Chelsea Fathauer, Ethan Rinne, Kandice K. Ludwig, Mary E. Lester, Ivan Hadad, Carla S. Fisher, Aladdin H. Hassanein

**Affiliations:** a Division of Plastic Surgery, Department of Surgery, Indiana University School of Medicine, 545 Barnhill Drive, Indianapolis, IN, USA; b Division of Breast Surgery, Department of Surgery, Indiana University School of Medicine, 545 Barnhill Drive, Indianapolis, IN, USA

**Keywords:** Aesthetic flat closure, Postmastectomy chest wall reconstruction, Going flat, Breast cancer, AFC

## Abstract

Postmastectomy patients may choose to not have breast reconstruction and opt for a flat closure. Aesthetic flat closure (AFC) is intentional incision selection and maneuvers for chest wall contour to optimize the aesthetic result. Patients may have a specific expectation of aesthetic outcomes, making AFC a new type of reconstruction. The purpose of this study is to perform a scoping review to define 1) patients who receive flat closure after mastectomy for treatment of breast cancer and their motivations for flat closure, 2) surgical techniques used to accomplish AFC, and 3) patient-reported outcomes of flat closure. A systematic search was conducted through MEDLINE, Embase, and Scopus. Articles were grouped into three overall categories of population description, surgical techniques, and outcomes. Within each category, major themes were identified. The literature search identified 48 articles and 15 met study criteria. Those who had no breast reconstruction considered risks, breast functionality, recovery time, and foreign body placement. Studies reported lack of surgeon support in patients’ decisions for flat closure. Surgical techniques for AFC included scar placement at the inframammary fold, medial and lateral debulking, and dog ear prevention. There were 89.9 % (328/365) of patients who were satisfied in choosing a flat closure and 58.2 % (689/1183) were satisfied with cosmesis. Patients chose flat closure for various reasons including avoiding additional surgery. Surgeon supported decision-making and comprehensive education on reconstructive and non-reconstructive options are critical to patient experiences. While patients are satisfied with their decision of flat closure, optimization of aesthetic results can be achieved by implementing chest wall reconstruction techniques with the specific goal of obtaining an AFC.

## Introduction

Breast cancer is the second-most common cause of cancer-related death in women.^1^ The incidence of breast cancer is reported to be over 2 million new cases worldwide.^2^ Breast reconstruction may be performed using implants or autologous tissue. There are a subset of patients who choose to forego breast reconstruction in favor of flat closure.^3^ This choice is colloquially referred to as “going flat.” A national database study of over 650,000 mastectomy patients reported 62.5 % underwent a flat closure.^4^

Although there are several approaches to closing the mastectomy site, the term “aesthetic flat closure” (AFC) is becoming more frequently used in the literature.^5^^,^^6^ AFC following mastectomy utilizes techniques that optimize scar positioning and contour of the chest wall.^5^ AFC is distinct from a flat closure following mastectomy without reconstruction. Patients may have a specific expectation of aesthetic outcomes, making AFC a new type of reconstruction.^5^ As a chest wall reconstruction, AFC differs from breast reconstruction by the lack of intention to recreate the appearance of breast tissue.

Plastic surgeons may more frequently be consulted for AFC. However, there is no standardized technique for AFC.^5^ The purpose of this scoping review is to characterize patients who undergo flat closure after mastectomy for treatment of breast cancer and motivations for flat closure in this context, AFC techniques to guide surgical decision-making, and patient-reported outcomes related to flat closure.

## Methods

A systematic search was conducted using MEDLINE, Embase, and Scopus databases. Search terms used in MEDLINE and Embase included “(mastectom*) AND ([flat adj5 closure] OR [“go* flat”] OR [aesthetic adj5 flat]).” Search terms used in Scopus were (ALL (mastectom*) AND ALL (“flat closure” OR “go flat” OR “going flat”). Articles were considered eligible if they presented primary research findings in any of the following three categories: population or motivations/influencing factors for flat closure (mastectomy without reconstruction), AFC surgical techniques, or patient-reported outcomes of patients who have undergone flat closure. Studies were excluded if patients had flat closure for reasons other than breast cancer treatment, only prophylactic mastectomy, or an intention of pursuing future breast reconstruction. Studies that were not primary research articles or not available in the English language were not included.

Two authors independently screened titles and abstract for relevance with a final full text review to select studies included in the final review. Two authors extracted data of interest from included studies. Data included author name, year of publication, and reported data related to the three predefined categories of population, surgical techniques, and outcomes. Patient-reported outcomes were further stratified into satisfaction with a decision for flat closure and satisfaction with cosmesis. Qualitative outcomes in the literature were quantified as response frequencies, which were pooled as the sum of response frequencies across studies to generate these patient-reported outcomes.

## Results

**Figure 1:**
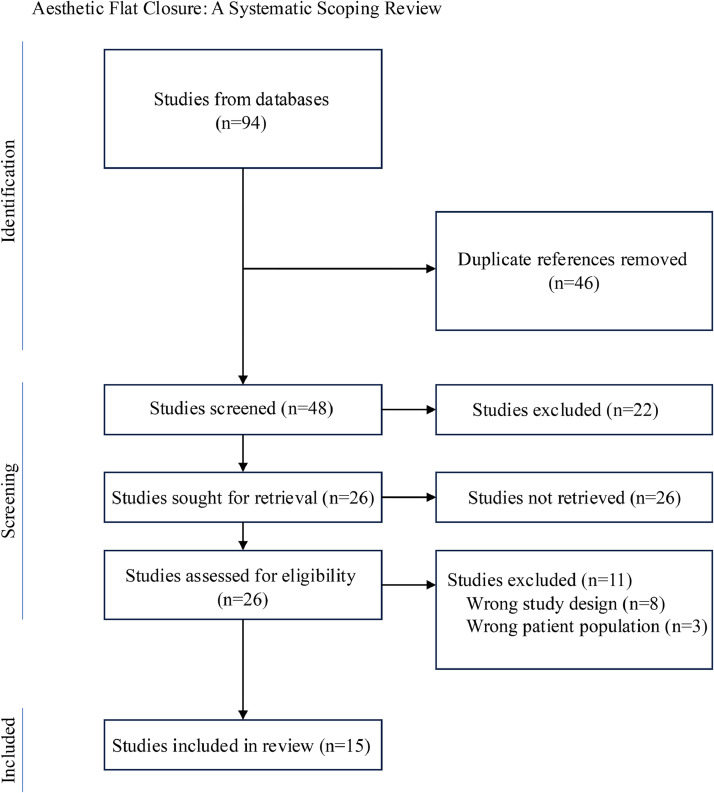
Visual representation of identification, screening, and inclusion of studies, with exclusion criteria. There were 15 studies that met inclusion criteria.

A total of 94 articles were assessed for eligibility. There were 46 duplicate studies which were removed. The remaining 48 studies were screened using inclusion and exclusion criteria, yielding 15 final studies relevant in our review (Fig. 1). These studies were further nonexclusively divided into three categories dependent on measures reported: population factors/motivations, surgical technique, and patient reported outcomes (Table 1, Table 2, Table 3, Table 4).

**Table 1:** Factors associated with choosing a flat closure.

Factor	Evidence
Age	• Patients who received flat closures were more often greater than or equal to 70 years of age at diagnosis (29% vs 4%; *p*<0.001)^4^ • Older patients were more likely to receive flat closure (*p*<0.001)^4^ • From 2004 to 2014 the rates of receiving flat closure steadily decreased for both women aged < 50 and in women aged 50–69. From 2015 to 2017, rates stabilized at 35 % for women aged < 50 and 56 % for women aged 50–69. From 2017 to 2019, the rates of flat closure increased in women < 50 from 35 to 38 %. Rates of flat closure remained stable for women ≥ 70 from 2014 to 2019 at approximately 88 %. Cochran-Armitage trend analysis was found to be significant for all years (*p*<0.001)^4^; Even amongst older patients, women aged > 75 were half as likely to undergo post mastectomy breast reconstruction compared with women aged 70–75 (*p*<0.001)^4^ • The patients undergoing reconstruction were younger than those who elected flat closure (median, 49 vs. 55 years; *p*<0.001)^7^
Race	• Patients who received flat closure were more often non-White than White race (25% vs 22%; *p*<0.001), • Patients who identified as non-Hispanic Black or Hispanic were more likely to receive flat closure than White patients (adjusted OR 1.37 and 1.17, 95% CI [1.33–1.40] and [1.13–1.21]; *p*<0.001)^4^
Insurance type	• A greater percentage of patients who received flat closures had government-provided insurance (*p*<0.001) • Women with either no insurance or government provided insurance were more likely to receive flat closures (*p*<0.001)^4^
Institution type	• Patients who received flat closures were more likely to receive surgery at a community cancer center or comprehensive community program compared with an academic/research program (adjusted OR 2.51 and 1.42, 95% CI [2.43–2.60] and [1.40–1.45]; *p*<0.001).^4^
Diagnosis-specific factors	• Patients who received flat closures had nodal involvement and higher stage disease (*p*<0.001)^4^ • Patients with a greater burden of local disease, defined as T4 tumors, were more likely to go flat (adjusted OR 2.82, 95% CI [2.48–3.20]; *p*<0.001)^4^ • Rate of flat closure was significantly lower with diagnosis of inflammatory breast cancer (*p*<0.001). Type of reconstruction (% non-inflammatory/% inflammatory): overall (43/57), delayed (47/’53), immediate (5.4/95), flat closure (39/61)^7^ • Receipt of both adjuvant chemotherapy and radiation therapy were associated with flat closure (adjusted OR 1.10 and 1.23, 95% CI [1.08–1.12] and [1.20–1.25); *p*<0.001, respectively)^4^
Comorbidities	• Patients who received flat closures more often had a Charlson-Deyo comorbidity score of ≥ 1 (20% vs 10%; *p*<0.001)^4^;
Sexual and gender minority (SGM) factors	• SGM breast cancer patients who chose flat closure (Flattoppers®) were more likely to have gone through treatment in the five years prior to taking the survey [completed between May 2015 and January 2016] (χ2 = 2.83, *p*<0.10)^8^ • Flattoppers® identified their sexual orientation as lesbian, bisexual, or queer and were more likely to have a genderqueer identity (χ2 = 8.67, *p*<0.05) or to identify their sexual orientation as queer (χ2 = 4.9, *p*<0.05)^8^ • Flattoppers® were significantly more likely to disclose to surgeons (χ2 = 5.25, *p* < 0.05), other physicians (χ2 = 4.27, *p*<0.05), and nurses (χ2 = 2.92, *p*<0.10)^8^ • Flattoppers® were more likely to report participating in an LGBT-specific support group or support network (χ2 = 7.51, *p*<0.01), whether in person or online^8^

**Table 2:** Motivations for flat closure.

Themes	Evidence
Practicality	• The top reason for choosing flat closure was desire for a faster recovery^6^ • When asked to describe their top two reasons for forgoing reconstruction, lower complication rate was the second most common reason, selected by 34.9 % of respondents^6^ • Some women associated age with breast functionality, and deemed that these factors precluded further use for a breast^11^ • For one younger participant, the additional surgery dissuaded her from the procedure. For her, breast surgery was more about survival than aesthetic^11^ • The women moved very quickly into a pragmatic, survival-focused mode after the initial shock of diagnosis: the breast became a sacrifice to eradicate any chance of the cancer remaining in their bodies, and the breast and cancer became synonymous. The women all described a pragmatic relationship with their breasts and towards breast reconstruction. Anya summarised, “you’ve still lost a breast whether you’ve got a reconstructed one or not”. The women’s drive to survive, recognition of the big picture and their understanding that replacing a breast cosmetically was a superficial augmentation of their body were clearly evident throughout their accounts.^10^ • They described various reasons for choosing flat closure, including avoiding the risks and complications associated with breast reconstruction, faster recovery times, and the need for fewer surgeries^9^ • Breast reconstruction surgery was “just not worth it”^9^
Avoidance of foreign material	• The second most common reason for choosing flat closure was avoidance of a foreign body placement^6^ • When asked to describe their top two reasons for forgoing reconstruction, avoidance of a foreign body was the most common reason, selected by 39.9 % of respondents^6^
Perception of “otherness” of reconstructed breasts	• The relationship with their own breasts as ‘natural’ directly contrasts with their expectations of breast reconstruction. Sarah said, ‘it looks like a breast but isn’t a breast’. This unnatural replacement was described in stark language highlighting its “otherness”^10^
Good friend/family experience or flat closure or bad experience of reconstruction	• They described various reasons for choosing flat closure, which included the influence of witnessing the positive or negative experiences of family or friends who went flat or had breast reconstruction^9^

**Table 3:** Surgical techniques for aesthetic flat closure.

Theme	Findings
Incision placement	• In the preoperative area, markings are performed in the upright position. Pitanguy's point (anterior transposition of inframammary fold along the midclavicular line) and the natural borders of the breast, including the inframammary fold are marked to confirm adequate length of the superior flap. A long gently sloping crescent shaped incision is drawn from a point 1–2 cm lateral to the sternum to the anterior axillary line, incorporating the nipple areolar complex, taking care to preserve as much skin and superior flap length as possible^12^ • The markings are made preoperatively by the plastic surgeon for the breast surgeon’s mastectomy incision to optimize the subsequent aesthetic flat reconstruction outcome. It is of utmost importance that the final incisions are symmetric bilaterally“^5^
Chest contour optimization	• The crescent incision and inframammary fold are scored and the lower flap is de-epithelialized, using epinephrine-soaked sponges to control dermal bleeding. In cases where the lower flap is relatively short, the infra-mammary fold can be divided to permit improved mobility of the lower flap for chest wall contouring. Redundant lateral tissue is de-epithelialized, advanced medially, and tacked to the pectoralis to smooth the side wall^12^ • The cranial mastectomy flap is allowed to overlap the caudal mastectomy flap to determine tissue redundancy. With the cranial flap overlying the caudal flap, excess tissue in the horizontal plane is assessed”^13^ • The thickness of the upper and lower breast flaps should be equal. A crucial component of the technique is thoughtful medial de-fatting on the chest wall to facilitate a smooth contour to the closure. In certain circumstances and with appropriate preoperative discussion, the best option to mitigate the presence of any dog ears can be a single excision with the incision from mid-axillary line to mid-axillary line utilized to provide a completely flat closure^5^ • Notably, a key intraoperative maneuver when performing these aesthetic flat closures is to sit the patient up on the operating room table to assess how the soft tissues re- drape when in a standing position. The plastic surgeon should be prepared to use staples to temporarily close, and to sit the patient up multiple times in the operating room until the proposed closure is entirely flat and linear^5^
Additional considerations: elevated BMI and excess tissue	• In the setting of elevated body mass index and significant subcutaneous fat in the upper abdomen, some relative concavity may result despite the chest wall contouring with the de-epithelialized flap and patients should be counseled appropriately. Similarly, lateral redundancy may persist despite de-epithelialization and medialization of the lateral tissue and require use of additional techniques targeting lateral dog ears, including liposuction, lateral extension of the incision, or excision of redundant skin paired with a “Y” or “fish-tail” plasty^12^ • To prevent the concavity observed in women with thick subcutaneous tissue in the epigastric region, the redundant caudal flap is de‐epithelialized to allow for some bulk^13^ • To optimize the aesthetic outcomes in obese patients, the following maneuvers are advised: (1) judicious lateral de-fatting is necessary to mitigate dog ears, specifically, aggressive lateral fat direct excision can be performed while ensuring that the flaps are not too thin; (2) axillary liposuction can be utilized to contour the lateral chest wall to provide smooth definition to the final flat chest closure; (3) tailor tacking is key in these patients in order to ensure that there is no lateral dog ear”; “the inferior flap should be elevated to the inframammary fold with careful attention to fully obliterate the inframammary fold by cauterizing below the inframammary fold^5^ • The IMF was then obliterated and undermined ∼ 10 cm to facilitate tissue advancement superiorly. Unilateral mastectomy surgery was performed in a similar fashion with the incision made just across midline to facilitate excision of the medial dogear^14^Excision of excess lateral tissue in the morbidly obese is more complex and requires additional considerations. While there have been numerous strategies described for this, Clough describes an approach where excess lateral skin and fat are excised through a vertical incision with subsequent medial advancement and thinning out of the lateral skin flap overlying the latissimus dorsi^14^ • Here, we want to describe a new versatile oncoplastic technique, the double S oncoplastic mastectomy, to avoid excessive axillary tissue and dog ears. It allows for resection of variable amounts of axillary skin and subcutaneous fat tissue and can be particularly useful in patients with lateral fat and skin abundance. Two small horizontal lines are drawn, one line above and one line under the nipple areola complex. These lines should mark the width of the skin segment to be resected and allow for tight skin closure. Then, two terminal landmarks of the incision are planned, one close to the xyphoid area and the other one in the anterior axillary line region. The final skin incision lines are then completed to delineate a double s-shaped segment. Both terminal points can be shifted medially and laterally in order to eliminate excessive skin and fat tissue if needed^15^
Additional considerations: breast size	• For women with a B cup or smaller breasts, the skin‐sparing mastectomy can generally be performed with a simple elliptical excision and closure. Any excess axillary tissue can be removed with a lateral extension to the incision. This permits a flat, aesthetically pleasing closure^13^ • Women with larger, more ptotic breasts require local tissue rearrangement to allow for a flat closure or to permit the creation of a breast mound^13^
Additional considerations: revision surgery	• The best revision procedures utilize bilateral breast flap advancements and judicious tissue excision. The reconstructive surgeon should be behooved to design an excision that will allow for an appropriate resection of the excess lower pole tissue, while balancing the need to close the wound by advancing the upper flap inferiorly to achieve a desired tight, flat closure^5^

**Table 4:** Patient-reported outcomes of satisfaction with choice and cosmesis of flat closure.

Patient outcomes	Satisfaction with choice (*n*=365)	Satisfaction with cosmesis (*n*=1183)
Baker et al. 2021	-	569/940
Everitt et al. 2023	-	-
Livingston–Rosanoff et al. 2020	232/250	92/109
Tyner et al. 2023	-	9/19
Wakeley et al. 2019	96/115	19/115
Total	328/365 (89.9%)	689/1183 (58.2%)

### Population/motivation

Factors significantly correlating with flat closure were older age, non-white race (non-Hispanic Black or Hispanic), increasing comorbidities (Charlson-Deyo comorbidity score of ≥1), government provided insurance or no insurance, location of treatment (community vs. academic/research institution), nodal involvement and higher stage disease (T4), treatment with adjuvant radiation therapy, treatment with adjuvant chemotherapy, and inflammatory type of breast cancer.^4^^,^^7^ (Table 1). Analysis by age determined that patients over 75 years of age are less than half as likely to undergo breast reconstruction following mastectomy as patients in the 70–75 age group.^4^ Bivariate analyses of 68 sexual and gender minority breast cancer patients found that patients who were diagnosed after 2011, identify as genderqueer, or participate in sexual and/or gender minority cancer support groups were associated with flat closure.^8^

Among those who elect for flat closure, motivations mainly include shorter recovery time, with both implant and autologous tissue, and avoidance of foreign body placement.^6^^,^^9^ (Table 2). Patients electing for flat closure over breast reconstruction also believed that there were lower complication risks associated with flat closure.^6^^,^^9^ On qualitative analysis of young breast cancer survivors (<50 years of age), reasons for choice of flat closure identified themes of practicality in opposition to reconstructed breasts.^6^ Practicality was rooted in 1) mastectomy without reconstruction as a route to eradicate chance of remaining cancer and 2) no longer needing breasts for functions such as breast feeding.^10^^,^^11^ Surveys of patients undergoing flat closure indicated that faster recovery times, fewer surgeries, and risks associated with breast reconstruction contributed to their decision to pursue a flat closure.^9^ Survey results also demonstrated that experiences of family and friends of patients contributed to their decision to pursue a flat closure compared to undergoing breast reconstruction.^9^

### Surgical technique

There were five studies that included surgical techniques of AFC.^5^^,^^12^, ^13^, ^14^, ^15^ AFCs are often longer and more complex procedures than traditional mastectomy with linear closure.^14^ The main differences lie in incision placement and optimizing the contour surrounding the mastectomy scar. There is no standardized AFC technique in the literature but there are common surgical principles to prioritize scar placement and contour optimization.

#### Incision placement

Scars can be optimized with strategic variations in position and length. Standard mastectomy removes the skin centered around the nipple-areola complex and thus places the scar above the inframammary fold. This can be used in a flat closure, but the scar falls in the middle of the pectoralis major muscle. Placement of the scar in an AFC has been described at the inframammary fold or the origin of the pectoralis muscle, which can be better concealed between the transition of the chest and abdomen.^5^

In addition to scar positioning, a single incision can be performed from mid-axillary line to mid-axillary line.^14^ Although this method is effective at creating a smooth medial contour between the left and right chest, a scar crossing the midline may be aesthetically unfavorable for patients.^5^ The scar is not well-concealed and skin over the sternum has a higher risk of keloid formation.^16^^,^^17^ Rather, a distinct sloped incision may be made bilaterally, from 1–2 cm lateral to the sternum medially to the anterior axillary line laterally.^12^ While two individual incisions are better concealed than the single incision, they must be carefully placed to appear symmetrical.^14^

#### Chest contour optimization

Removing central chest tissue can create standing contour deformities and accentuate excess skin and soft tissue, particularly medially near the sternum and laterally in the subaxillary area.^15^ Without the breast mound on the chest, these pre-existing areas are now in full anterior view, easily seen by the patient.^6^ This is most pronounced in obese patients.^12^^,^^14^ Debulking the subcutaneous soft tissue medially and laterally can be performed with direct excision through the incision.^15^ Alternatively, medial chest and axillary liposuction improve the smooth and flat appearance of an AFC, debulking the subcutaneous tissue without extending the incision.^5^^,^^12^

A single incision method can facilitate removal of excess tissue in the midline.^14^ With distinct incisions, curving the incision upwards at the medial edge before the sternum helps keep two distinct scars while minimizing medial dog-earing.^13^^,^^14^ The incision extended laterally into the subaxillary area removes excess lateral soft tissue.^15^ To further address excess lateral chest wall tissue in obese patients, a vertical incision overlaying the latissimus dorsi may be made with subsequent medial advancement to further reduce lateral skin and fat.^14^ Alternatively, an S-shaped incision within the vertical boundaries of the upper and lower nipple line and terminal boundaries of the xyphoid and anterior axillary line reduces excess medial and lateral chest wall tissue.^15^ This procedure decreases the size and tension of the incision compared to a lateral L shaped incision.^15^

Another important technical consideration is obliteration of the inframammary fold for a smooth transition between the chest and abdomen. This technique minimizes excess skin and standing cone deformities.^5^ Cauterization of the fascia below the inframammary fold provides laxity to elevate the superior and inferior skin flaps.^5^ In obese patients, the length of cauterization may reach about 10 cm.^14^ Liposuction of the inframammary fold can also achieve a smooth contour between the chest and abdomen.^5^

Chest wall comesis may be optimized by avoiding size mismatch and thickness of superior and inferior skin flaps.^5^ Chest wall concavity can further be alleviated by de-epithelializing the inferior flap, securing the tissue to the pectoralis, and laying the superior flap over the de-epithelialized inferior flap.^12^^,^^13^ This method also reduces skin redundancy.^5^^,^^13^ The chest volume and thickness will likely still be deficient, but the transition between chest and abdomen will be more subtle. In the case of a short inferior mastectomy skin flap, the de-epithelialized flap can be divided to mobilize volume for chest wall contouring.^12^ A de-epithelialized lateral mastectomy skin flap can be medially mobilized and secured to the pectoralis to smoothen the lateral aspect of the chest wall.^12^Intraoperative adjustment to a sitting position is a straightforward approach to identify excess tissue.^5^

In some cases, excess skin in the horizontal plane is removed with a small lateral incision similar to a Wise pattern closure.^13^ The Wise pattern type of closure gives a horizontal and vertical scar and is used in the Goldilocks procedure, which gives a small central mound with de-epithelialized, buried inferiorly based dermal skin.^18^^,^^19^ However, the Wise pattern is meant to project centrally by creating a vertical standing cone.^20^^,^^21^ For a patient who desires no appearance of a breast, this maneuver may not be desirable. Further, without a breast, the vertical limb of the Wise pattern is perpendicular to the relaxed skin tension lines and can be prominent.^22^ Additionally, the triple-point may adversely affect wound healing.^23^

#### Additional considerations

Patient habitus should be considered in surgical planning. Patients with high BMI should be adequately counseled regarding chest wall concavity and excess lateral skin despite measures taken to reduce these.^12^ Skin-sparing mastectomy with elliptical excision can be modified with removal of excess lateral axillary tissue with lateral excision extension to create a flat closure with good surrounding contour for patients with smaller breasts and low body mass index (BMI).^13^ On the other hand, large, ptotic breasts in an obese patient may require additional maneuvers for optimal flat aesthetic closure.^13^ Revisions of prior mastectomies generally concern excess medial or lower pole chest wall tissue which can be remedied by tissue excision of the lower pole and maneuvering the upper flap inferiorly to balance, tighten, and flatten the closure.^5^ Patients with prior radiation are at elevated risk for skin necrosis or wound healing problems and should be counselled about this.^24^

### Patient-reported outcomes for flat closure

Published patient-reported outcomes are available for flat closures, but specifically for AFC. A study of 931 patients performed by Baker et al. showed that patients who underwent flat closure endorsed an 83.5 % satisfaction rate.^6^ In patients who underwent surgery for ductal carcinoma in situ, the odds ratio for answering “somewhat satisfied” in comparison to “very satisfied” was OR 2.77 CI 1.24–6.24 for autologous vs. no breast reconstruction.^25^ This data indicates that flat closure patients were more likely to choose very satisfied over satisfied than their breast reconstruction counterparts. Satisfaction with choice of flat closure was calculated based on patient survey responses of “I believe I made the right decision for me” or “very satisfied”/“somewhat satisfied” with their surgical decision. Patient satisfaction with surgery may be a combination of perception of functionality, quality of life, recovery, support, and other factors. Aggregation of patient outcomes revealed that 89.9 % of flat closure patients were satisfied with their choice of surgery (Table 4).

While patients were satisfied with their decision, satisfaction with cosmesis of flat closure ranged. Satisfaction with cosmesis was determined by survey responses to the statements “I am pleased with the appearance of my chest,” “Overall I am satisfied with my surgical outcome,” or “I feel happy, satisfied, or grateful for my results,” or by verbal endorsement of satisfaction or dissatisfaction via Zoom interview or during follow up. Satisfaction with cosmesis reflects aesthetic expectations and outcomes. There were 58.2 % of patients satisfied with cosmesis of their flat closure, revealing a glaring disparity between happiness with surgical decision and satisfaction with results. Wound healing risk factors and clinical complications (ex. seroma, infection) were not associated with cosmetic concerns.^26^

## Discussion

AFC as a term is increasing in popularity.^5^ Our scoping review indicates that the term is heterogenous, with multiple techniques which vary in scar placement, number of scars, and strategies to improve contour surrounding the incision.^5^^,^^12^, ^13^, ^14^, ^15^ At minimum, AFC is distinct from a standard simple mastectomy linear closure by attempts to optimize scar placement and to minimize standing cone deformities medially and laterally. Patient satisfaction with choosing flat closure after mastectomy for treatment of breast cancer was high, at 89.9 % (328/365) while satisfaction with cosmesis of the flat closure received was only 58.2 % (689/1183).

Factors associated with choosing flat closure over breast reconstruction include race, older age, comorbidities, having government insurance or no insurance, type of institution at which treatment occurred (academic versus community), stage of breast cancer, and adjuvant radiation or chemotherapy.^4^^,^^7^^,^^8^ Motivations for receiving mastectomy without breast reconstruction included lower complication risks and recovery time, views of breast reconstruction as unnatural, and familiarity with a family or friend’s positive experience with flat closure or negative experience with breast reconstruction.^6^^,^^9^^,^^10^ In patients who have undergone flat closure, lower satisfaction scores are correlated with decreased surgeon support, higher BMI (>30kg/m^2^), and unilateral procedure.^6^ Dissatisfaction with cosmesis was reported with unmet aesthetic expectations, such as standing cone deformities and postoperative scars.^27^ Satisfaction with flat closure is high in women who choose this procedure, though support systems and surgical procedures can be developed to further facilitate decision making and optimize aesthetic surgical outcomes.^6^^,^^9^^,^^25^, ^26^, ^27^

Between 2013 and 2019, only 43 % of patient who underwent mastectomy to treat breast cancer also underwent breast reconstruction.^28^ After the Women’s Health and Cancer Rights Act of 1998 encouraged years of successively increasing reconstruction rates, rates of reconstruction have stabilized since 2014.^29^, ^30^, ^31^ The flat closure rate has particularly stabilized in patients less than 70 years of age, and shifts in momentum, such as the “going flat” movement, are increasing publicity of flat closures.^4^ With survivorship increasing and breast cancer patients benefiting from more effective therapies and longer lives than in previous decades, patient motivations and satisfaction are important to understand and incorporate into treatment.^32^

One of the most important steps in surgical counseling is to understand the patient’s surgical goals and find the treatment plan that best suits their goals. Patient expectations should be elicited through conversation and/or visual examples, and realistic outcomes should be discussed. Predicting the look or how a patient will feel without breasts was a key influential factor noted in a qualitative study of patients’ decision-making process for mastectomy with or without reconstruction.^11^ Additionally, expectations should be managed by communicating the potential of revisions.^13^ During surgical consultation, it is important to ask about patient goals, particularly about how they envision their flat closure.^5^ It should be made clear that a flat closure may actually appear concave, relative to the abdomen depending on the body habitus. In patients undergoing flat closure, adequate counseling about surgical options and having a surgeon specializing in breast surgery was correlated with greater satisfaction with surgical outcome.^6^

**Figure 2:**
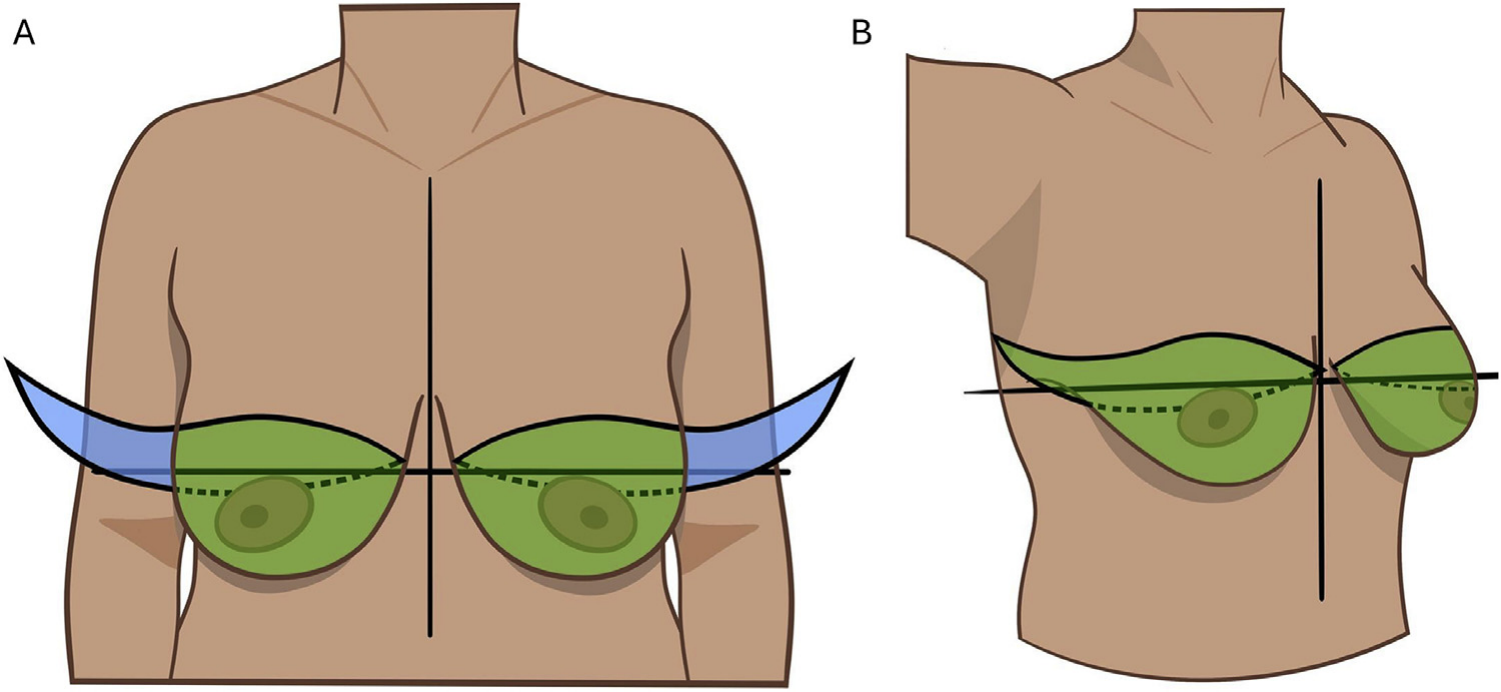
Illustration of an aesthetic flat closure. (A) The scar may be placed at the inframammary fold (IMF) rather than at the central chest to optimally conceal the scar between the transition of the chest and abdomen. This involves marking the inframammary fold in the preoperative area while the patient is standing and an ellipse on the central breast skin at Pitanguy’s point transposed anteriorly on the breast skin. The superior skin resection can be modified intraoperatively based on skin excess. Minimizing standing cone deformity of the medial chest can be achieved by curving the incision slightly superiorly and directly de-fatting the subcutaneous adipose at the edge of the scar. Liposuction is utilized medially to minimize the dog-ear, across the subcutaneous tissue over the sternal area in obese patients, transcending the inframammary fold to minimize a prominent soft tissue fold, and laterally in the subaxillary area bilaterally. (B) The lateral aspect of the excision is extended into the subaxillary area with a slight superior curve to remove excess lateral skin.

**Figure 3:**
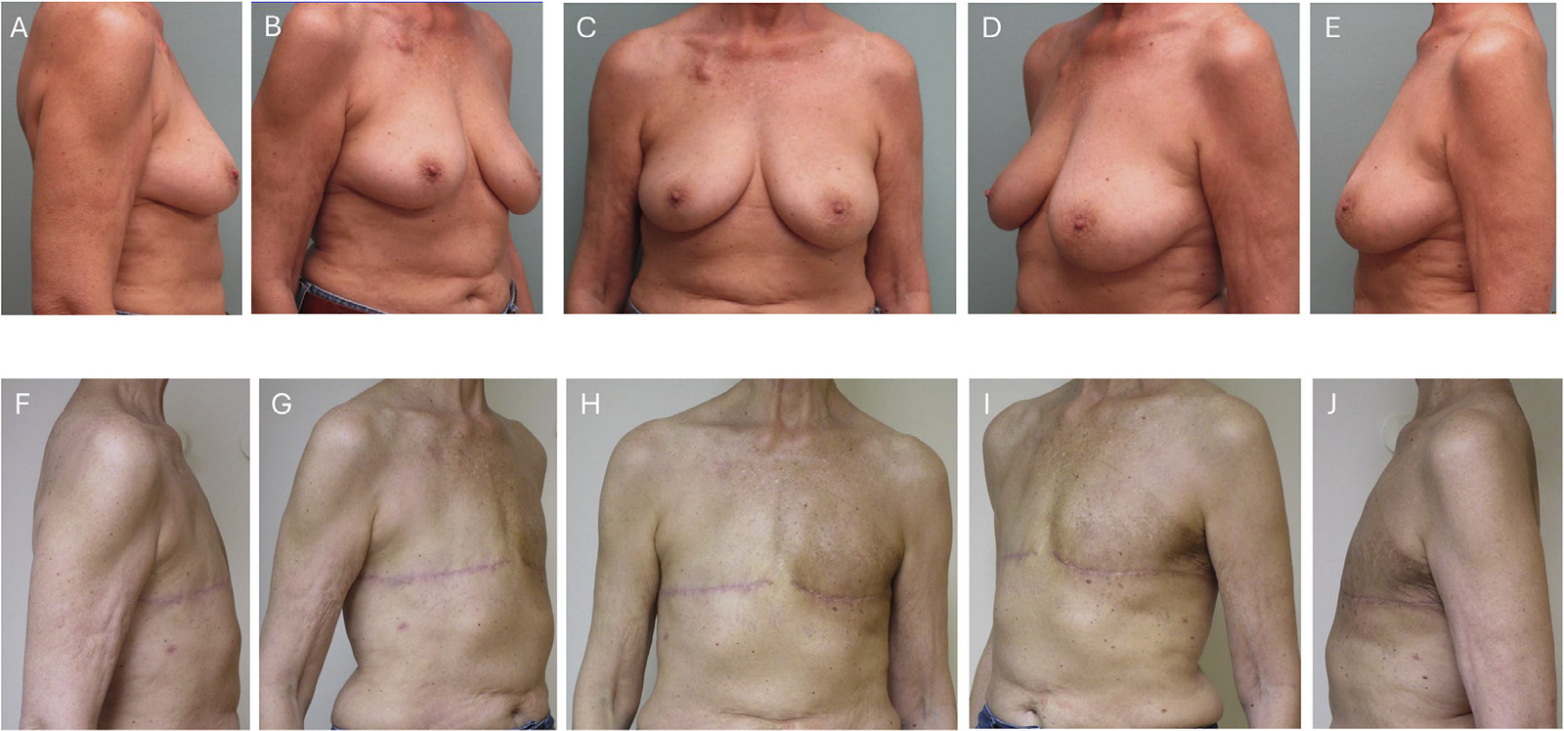
62-year-old female with left breast cancer who requested bilateral mastectomy and aesthetic flat closure. (A–E) Top panels exhibit pre-operative views. (F–J) Bottom panels show 5 months postoperatively and following adjuvant radiation.

**Figure 4:**
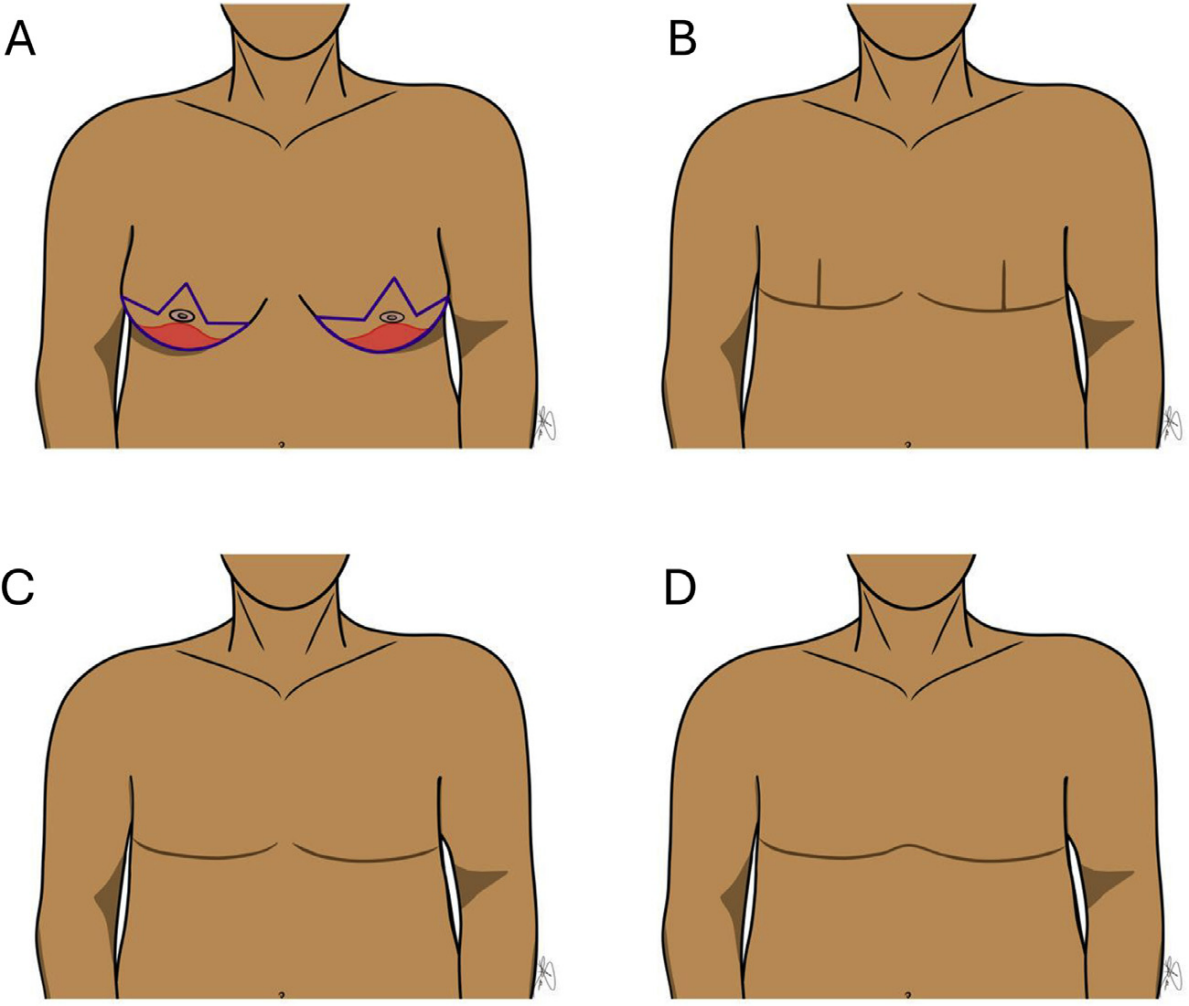
Flat closure approaches. (A) Goldilocks procedure using Wise pattern type of markings and utilizing an inferiorly based de-epithelialized dermal skin flap which is buried under the closure over the pectoralis major muscle. (B) Inverted-T scar of the Goldilocks procedure. (C) Aesthetic flat closure scar with scars at the inframammary fold. (D) Alternative aesthetic flat closure technique connecting the scar across the midline.

Although there are variations in techniques for AFC (Table 3), we have a favored approach based on the principles of the scoping review and the senior author’s (AHH) preference (Supplemental Table 1). The scar placed at the inframammary fold rather than at the central chest optimally conceals the scar between the transition of the chest and abdomen. The inframammary fold should be marked in the preoperative area with the patient standing. At this time, an ellipse should be marked on the central breast skin at Pitanguy’s point transposed anteriorly on the breast (Fig. 2). The superior skin should be assessed in the intraoperative sitting up position and modified based on skin excess. The standing cone deformity of the medial chest is minimized by curving the incision slightly superiorly and directly de-fatting the subcutaneous adipose at the medial edge of the scar. Liposuction is utilized medially to minimize the dog-ear, across the subcutaneous tissue over the sternal area in obese patients, transcending the inframammary fold to minimize a prominent soft tissue fold, and laterally in the subaxillary area bilaterally. The lateral aspect of the excision is extended into the subaxillary area with a slight superior curve to remove excess lateral skin (Fig. 3). The decision between a classic Goldilocks procedure, with de-epithelialization of the inferiorly based dermal flap with Wise pattern inverted T closure, versus true AFC is patient dependent. In patients who prefer a small projected mound and are accepting of an additional vertical scar in each side of the chest, the Goldilocks procedure may be preferred (Fig. 4).

In a social media survey study of breast cancer survivors in “go flat” groups, over a third indicated that they would have preferred physicians to include flat closure as a treatment option and 30.6 % specifically wanted providers to include flat closure as a part of standard surgical counseling.^27^ Bias from healthcare teams towards breast reconstruction has been noted by patients who elect to undergo flat closure, especially by younger patients. Some patients endorse experiencing “flat denial,” in which they are left with excess soft tissue after mastectomy against their stated desire for a flat closure, negatively affecting aesthetic outcomes. Rates of flat denial in the literature surpass 20 %.^12^ In these situations, some patients report that flat closure is not supported as a treatment plan. Patients who choose AFC, particularly younger patients, report perceived pressure to reconsider their choice.^10^ With breast reconstruction being seen as the norm and default for younger patients, these patients feel marginalized for choosing flat closure.^9^^,^^10^ Qualitative survey responses from lesbian patients and individuals that do not identify as female gender reveal the theme that disclosing sexual orientation increases providers’ support for their decision for flat closure.^8^ Comparisons between gender-affirming surgery and AFC in the post-oncological setting can be found in Appendix 1.

This study had limitations pertaining to the nature of reviewing published literature. The quality of published literature allowed for only a scoping review. As a scoping review, this study is limited only to the findings presented by existing primary literature articles. Reasons of patients receiving a flat closure may be heterogenous. In our scoping review, we identified studies in which patients received flat closure.^6^^,^^9^^,^^25^, ^26^, ^27^ In the published data on flat closures, the subset receiving AFC specifically is not quantified.^6^^,^^9^^,^^25^, ^26^, ^27^ In many of the studies, patient’s choice in receiving the flat closure is implied, such as survey questions asking about satisfaction with patients’ decision of flat closure surgery, limiting the ability to specifically quantify patient outcomes of AFC and serving as a potential confound.^6^^,^^9^^,^^25^, ^26^, ^27^ However, studies rarely confirm election of flat closure as an inclusion criteria. Among included studies, delineation of a subset of patients who received a flat closure (mastectomy without reconstruction) because of lack of access, not appropriate candidates for breast reconstruction, or awaiting future reconstruction was not clear. There were limitations to assess patient satisfaction as the included studies did not use formal validated tools to evaluate patient satisfaction with their decisions and their outcomes. Further multicenter, prospective studies with a validated, standardized measure quantifying patient responses using a Likert scale may be beneficial to the characterization of patient reported satisfaction with flat closures. As we move towards standardizing the AFC, such a measure would be valuable in measuring progress in patient reported satisfaction. Additionally, there may be research articles that were not included in review that include relevant information and perspectives. However, due to the robust nature of the multi-database literature search using MeSH terms and accordance with the PRISMA-ScR Checklist, we believe that the included articles are representative of the published data on flat closure following mastectomy for treatment of breast cancer.

## Conclusion

AFC is an option for patients who do not desire breast reconstruction. AFC is distinct from direct linear closure for simple mastectomy. Although no standardized AFC technique exists, scar placement and contour optimization are common aspects. This scoping review aimed to provide greater understanding of populations and motivations of women with breast cancer who undergo mastectomy with flat closure, surgical techniques used to achieve an AFC, and patient reported outcomes related to flat closure. While patients are satisfied with their choice to remain flat, aesthetic flat closure techniques may improve satisfaction.

## Ethical approval

This study did not involve human participants and does not require ethics committee approval.

## Data availability statement

The data supporting the findings are available from the corresponding author upon reasonable request.

## Declaration of competing interest

The authors declare that they have no known competing financial interests or personal relationships that could have appeared to influence the work reported in this paper.

## Appendix

Along the spectrum of flat closures are transgender patients who benefit from gender affirming mastectomy. Although this is not part of the studies included in this scoping review, those patients may require a different approach than AFC. It is important to establish the specific goals of the patient in this situation as patients may be non-binary. In a standard masculinizing top surgery, the skin resection inferiorly begins at the IMF and the height of the resection superiorly is at Pitanguy’s point.^33^, ^34^, ^35^, ^36^ It is paramount that the scar lands at or slightly below the pectoralis.^34^^,^^37^^,^^38^ There should be zero tension on the closure, as these highly visible scars tend to widen or hypertrophy over time.^34^ The incisions start medially about 1-2 cm from the midline, and travel in a gentle curve laterally.^39^ Laterally, the apex of the incision heads cephalad, toward the mid axillary line.^39^ The pectoralis fascia is not removed and the chest flap of remaining skin leaves breast tissue *in situ*.^34^ Normally, the upper flap of tissue is left at least as thick as the cut edge of skin and fat at the IMF.^38^ Doing so creates an actual flat closure. Generally, AFCs after true mastectomy are actually concave on lateral view.^5^ This deformity can later be mitigated with fat grafting or male pectoral silicone implants.^40^

Liposuction or direct excision medially along the sternum and laterally beyond the skin incisions is key to help flatten the periphery of the chest.^41^ Liposuction in the axilla/tail of Spence area is also often key to creating an aesthetic pectoral groove.^42^ The nipple usually thinned out into an oval full thickness skin graft and replaced onto the closed chest.^42^ This is grafted onto a “traditionally masculine” position, but size, shape and position can be modified to patient preferences.^41^^,^^42^

**Appendix 1.** Comparisons between gender affirming surgery and AFC in the post-oncologic setting.

## Appendix Supplementary materials

Image, application 1
