# Supplementary material for: Evaluation of aesthetic flat closure: A scoping review
Source: JPRAS Open. 2025 Aug 24;46:69–82. doi: 10.1016/j.jpra.2025.08.024 (PMC12596527; doi:10.1016/j.jpra.2025.08.024)
Supplement: Supplementary file 1 [file mmc1.docx]

*Journal of Plastic, Reconstructive & Aesthetic Surgery Open* Review Article

**Supplemental Table 1:** Aesthetic Flat Closure Steps.

| **Step** | **Description** |
| --- | --- |
| 1: | Mark the patient preoperatively, with the patient standing. Mark the following basic breast landmarks: sternum, IMF, anterior axillary line, and breast meridian. |
| 2: | Mark Pitanguy's point by transposing the IMF location at breast meridian anteriorly to the breast. Design an ellipse with Pitanguy's point as the peak and medial limit lateral to the ipsilateral sternum edge. Laterally, extend to the subaxillary region to allow for removal of excess lateral skin along the IMF. |
| 3: | Incise along the inferior mark of the marked ellipse. |
| 4: | Dissect adipocutaneous flaps over the pectoralis. |
| 5: | Taylor tack prior to commitment to superior incision of the ellipse to adjust as needed for a closure that allows for adequate removal skin without excessive tension. |
| 6: | Conduct suction assisted lipectomy, laterally in the subaxillary region beyond the skin excision and medially at subcutaneous tissue over the sternum. |
| 7: | Surgically disrupt the IMF to allow for redraping of flaps. |
| 8: | Install a drain and close the surgical site. |
